# Thyroid hormone metabolism and environmental chemical exposure

**DOI:** 10.1186/1476-069X-11-S1-S10

**Published:** 2012-06-28

**Authors:** Marike M Leijs, Gavin W ten Tusscher, Kees Olie, Tom van Teunenbroek, Wim MC van Aalderen, Pim de Voogt, Tom Vulsma, Alena Bartonova, Martin Krayer von Krauss, Claudia Mosoiu, Horacio Riojas-Rodriguez, Gemma Calamandrei, Janna G Koppe

**Affiliations:** 1 Department of Paediatrics and Neonatology, Emma Children’s Hospital Academic Medical Centre, Amsterdam, The Netherlands; 2 IBED/ESS, University of Amsterdam, Amsterdam, The Netherlands; 3 University Hospital Aachen RWTH, Department of Dermatology, Pauwelstrasse 30, 52074 Aachen, Germany; 4 Department of Paediatrics and Neonatology, Westfriesgasthuis, Maelsonstraat 3, 1624 NP Hoorn, The Netherlands; 5 KWR Watercycle Research, POBox 1072, 3430 BB Nieuwegein, The Netherlands; 6 Ministry of Housing, Spatial Planning and the Environment, The Hague, The Netherlands; 7 NILU – Norwegian Institute for Air Research, Kjeller, Norway; 8 WHO, Regional Office for Europe,Copenhgen, Scherfigsvej 8, Denmark; 9 Institute of Food Bioresources (IBA), Bucharest, Romania; 10 National Institute of Public Health, Cernavaca, Morelos, Mexico; 11 Istituto Superiore di Sanita, Rome, Italy; 12 Ecobaby Foundation, Hollandstraat 6, 3634 AT Loenersloot, The Netherlands

## Abstract

**Background:**

Polychlorinated dioxins and –furans (PCDD/Fs) and polychlorinated-biphenyls (PCBs) are environmental toxicants that have been proven to influence thyroid metabolism both in animal studies and in human beings. In recent years polybrominated diphenyl ethers (PBDEs) also have been found to have a negative influence on thyroid hormone metabolism. The lower brominated flame retardants are now banned in the EU, however higher brominated decabromo-diphenyl ether (DBDE) and the brominated flame retardant hexabromocyclododecane (HBCD) are not yet banned. They too can negatively influence thyroid hormone metabolism. An additional brominated flame retardant that is still in use is tetrabromobisphenol-A (TBBPA), which has also been shown to influence thyroid hormone metabolism.

Influences of brominated flame retardants, PCDD/F’s and dioxin like-PCBs (dl-PCB’s) on thyroid hormone metabolism in adolescence in the Netherlands will be presented in this study and determined if there are reasons for concern to human health for these toxins. In the period 1987-1991, a cohort of mother-baby pairs was formed in order to detect abnormalities in relation to dioxin levels in the perinatal period. The study demonstrated that PCDD/Fs were found around the time of birth, suggesting a modulation of the setpoint of thyroid hormone metabolism with a higher 3,3’, 5,5’tetrathyroxine (T4) levels and an increased thyroid stimulating hormone (TSH). While the same serum thyroid hormone tests (- TSH and T4) were again normal by 2 years of age and were still normal at 8-12 years, adolescence is a period with extra stress on thyroid hormone metabolism. Therefore we measured serum levels of TSH, T4, 3,3’,5- triiodothyronine (T3), free T4 (FT4), antibodies and thyroxine-binding globulin (TBG) in our adolescent cohort.

**Methods:**

Vena puncture was performed to obtain samples for the measurement of thyroid hormone metabolism related parameters and the current serum dioxin (PCDD/Fs), PCB and PBDE levels.

**Results:**

The current levels of T3 were positively correlated to BDE-99. A positive trend with FT4 and BDE-99 was also seen, while a positive correlation with T3 and dl-PCB was also seen. No correlation with TBG was seen for any of the contaminants. Neither the prenatal nor the current PCDD/F levels showed a relationship with the thyroid parameters in this relatively small group.

**Conclusion:**

Once again the thyroid hormone metabolism (an increase in T3) seems to have been influenced by current background levels of common environmental contaminants: dl-PCBs and BDE-99. T3 is a product of target organs and abnormalities might indicate effects on hormone transporters and could cause pathology. While the influence on T3 levels may have been compensated, because the adolescents functioned normal at the time of the study period, it is questionable if this compensation is enough for all organs depending on thyroid hormones.

## Background

Polychlorinated biphenyls (PCBs) and dioxins (PCDDs/Fs), especially TCDD (2,3,7,8-tetrachloro-dibenzo-p-dioxin) are well known developmental endocrine disruptors. PCDDs/Fs and planar (dioxin-like, dl-) PCBs are often grouped together as ‘dioxins’ or ‘dioxin-like compounds’, because of their common mode of (toxic) action, via the Ah-receptor.

PCDDs and PCDFs are unwanted by-products of the production of chlorinated phenols, metallurgic processes, bleaching of paper pulp and the incineration of waste [1-3]. PCBs have been produced world-wide from the 1930s and were mainly used as dielectric fluids in electrical transformers and capacitors, as heat exchange or hydraulic fluids [4].

The polybrominated diphenylethers (PBDEs) have been widely used over the last few decades as flame retardants in various materials such as electronic equipment, plastics, foams (e.g. used in car seats and furniture), carpet liners and textiles. Humans are exposed to PBDEs mainly by ingestion (from food and milk) and by inhalation of indoor air and dust. Currently, these compounds are frequently detected in humans all over the world [5,6]. The penta- and octa brominated diphenylethers are banned in the European Union, but are still present in the environment. Furthermore, three others, also blamed for interfering with thyroid hormone metabolism are still in use, decabromo diphenyl ether (DBDE), hexabromocyclododecane (HBCD), and tetrabromobisphenol A (TBBPA). The first two were extensively addressed in the HENVINET project. This FP6 EU HENVINET project aimed at synthesizing scientific information available on a number of topics of high relevance to researchers and policy makers in the field of environment and health (E.C. grant: HENVINET 037019). The third one TBBPA is currently found in human beings and can traverse the placenta as it has been found in babies born by caesarean section in hospitals. [7] TBBPA has a half life of 2 days and is excreted as a glucuronide or a sulphate in the faeces via the bile. In animal studies effects are detected on the apical part of the cochlea and an increase in pituitary weight was observed [8,9]. The specific thyroid hormone nuclear receptor TR- ß2, only present in the cochlea, pituitary gland and hypothalamus might play a role. Considering the findings in babies born in hospital the margin of exposure is very low and current use of TBBPA is therefore a matter of concern for human health, especially in the perinatal period. Thyroid hormone is essential for normal body metabolism, growth, and development including reproduction, maturation and ageing. Fluctuations in thyroid hormone levels are able to alter outcomes in children [10,11].

Large amounts of these compounds have been released into the environment through the processes previously stated. Organisms, and ultimately humans, are exposed via ingestion (food, drinking water), via inhalation, and via dermal contact. Ingestion is the main source (90%) of exposure, primarily through meat and meat products (23-27%), dairy products (17-27%) and fish (16-26%) [12]. Due to the accumulating properties of these compounds, each step higher in the food chain increases the concentration of dioxins in an organism (bioaccumulation). Once ingested, dioxins and PCBs are primarily stored in the liver followed by the adipose tissue. After ingestion dioxins and PCBs are detectable for a long period. The mean half-life of dioxins and PCBs in the human body is assumed to be 7 to 9 years [13].

Dioxins, PCBs and PBDE’s are also able to cross the placenta[14] . In addition, they are excreted in breast milk and thereby cause significant exposure to nursing offspring [15]. Adolescents, undergoing hormonal changes during puberty, are probably also at greater risk of susceptibility, and therefore at higher risk, with regards to environmental exposure health effects [16].

Background concentrations, concentrations that average individuals in Europe and the US are daily exposed to, have been related to various negative health effects. Studies have shown negative effects on lung function [17,18], and haematological and immunological disturbances [19-21]. In addition, an increase in behavioural problems was seen, and prolonged evoked responses measured with EEG (electro-encephalography) and MEG (magneto-encephalography), and possible indications of subtle neurological abnormalities were found in children [22-25].

Previous studies of the current cohort showed a higher T4/TBG ratio that became significantly higher at 7 days and 11 weeks after birth, together with an increase in TSH. This finding was interpreted as an hypothyroidal state of the hypothalamic cells involved in thyroid hormone metabolism caused by dioxins [26].

In this study thyroid hormone parameters were investigated in relation to prenatal and early postnatal PCDD/F exposure and current exposure of PCDD/F and dl-PCBs and the lower brominated PBDE’s during adolescence.

## Methods

### Study population

This study is part of a longitudinal cohort study of 14-19 year old children, studied during their neonatal (n=60), [27] toddler (n=60) [28] and pre-pubertal period (n=41) [22]. All 33 children (18 girls and 15 boys) participating in the current follow-up were born in the Amsterdam/Zaandam region. Twenty-five of the children are still inhabitants of the region. PCDD/F exposure was determined in the perinatal period in breast milk. Of the total cohort of 41 subjects who participated in the pre-pubertal study, one subject was excluded from the current follow-up because of a Ewing sarcoma and one was partly excluded because of an extra Y-chromosome (XYY). Five subjects declined to participate in the new follow-up, three could not be traced. One of the children, who did not participate in the pre-pubertal follow-up, consented to the current follow up. Of the 33 examined adolescents 2 refused to undergo vena puncture and 2 refused a repeated puncture after blood clotting in the first needle.

The study was approved by the institutional medical ethics committee. All participants of the study and their parents signed an informed consent.

### Laboratory analyses

Perinatal PCDD/F levels and current serum levels of PCDD/Fs, dl-PCBs and PBDEs were measured in an uncontaminated laboratory dedicated to low-level dioxin sample treatment, at the Environmental Chemistry Section of IBED/ESS of the University of Amsterdam. Concentrations of the 19 most toxic PCDD/F congeners (seven PCDDs and twelve PCDFs) and the concentration of 3 dl-PCBs (77, 126, 169) and 8 PBDEs (28, 47, 85, 99, 100, 153, 154 and 183) were determined. The concentration of PCDD/F and dl-PCB congeners are expressed in toxic equivalents (TEQ) ng/kg=pg/g fat.

An activated carbon column (Carbosphere) was used for group separation of the chemicals. The PCDD/F and dl-PCB fraction was isolated and a clean-up was performed using a column of AgNO3 on silica gel and a column of activated Al2O3 on silica gel. The PBDE fraction was purified using activated Al2O3 on silica gel and an activated alumina column. After concentrating the sample, quantification of dioxins and dl-PCBs was done using hr-GC/hr-MS. PBDEs were determined by hr-GC/lr-MS. As an internal standard, a mixture of 13C-labelled PCDD/Fs, dl-PCBs and PBDEs was used. More detailed information about the analysis have been published elsewhere [6].

PCDD/F concentrations were previously determined in the mothers’ milk 3-4 weeks after birth, which is indicative of the prenatal exposure. The cumulative total postnatal/lactational exposure was calculated as the measured PCDD/F concentration in breast milk multiplied by the total breast milk intake. [29] Results see table 1.

**Table 1:** Dioxin, dl-PCB and PBDE exposure

	Mean	Range	Standard deviation	95% confidence interval
Prenatal dioxin (PCDD/F) exposure ITEQ (pg/g lipid in breastmilk), n=32	32.6	9.05-88.8	64.3	25.9-38.5

Lactational dioxin (PCDD/F) exposure ITEQ (absolute quantity in ng), n=32	66.5	4.34-279	64.3	43.3-89.6

Current serum dioxin (PCDD/F) WHO-TEQ (pg/g lipid in serum), n=27	2.2	0.4-6.1	1.6	1.6-2.8

Current serum dl-PCBs WHO-TEQ (pg/g lipid)	2.2	0.04-7.8	2.0	1.4-3.0

Current serum PBDE (ng/g lipid) n=17*	10.5	4.9-22.1	4.6	8.2-12.9

Means, ranges, standard deviation and 95% confidence interval of the exposure to Dioxins, PCBs and PBDE’s.

*Current serum PBDE levels is calculated with exclusion of the outlier (73.6 ng/g lipids)

### Statistical analyses

For statistical analyses the non-parametric Spearman’s correlation coefficient was calculated using SPSS- 14.0. The level of significance was 5% (P=0.05) for the analysis with the predicted variables. For the congener specific analysis the level of significance was 5/8% (P=0.0063), to correct for the number of analyses.

As outcome variables we used serum T3, T4, FT4, TSH and TBG. The prenatal, lactational and current serum PCDD/Fs and the current serum dl-PCBs and ∑PBDE levels were the predicted variables using the Spearman’s correlation coefficient. A congener specific analysis of the PBDEs was performed.

## Results

### PCDDs/Fs

No correlations were found between prenatal dioxin exposure and T3 (P=0.14), T4 (P=0.16), FT4 (P=0.81) TBG (P=0.25) or TSH (P=0.78). Neither was a correlation seen with the lactational exposure. Means and ranges are given in table 2.

**Table 2:** Thyroid hormone metabolism parameters

*Measured objective n*=*29*	Mean	Range	Standard deviation	95% confidence interval
Free thyroxin (FT4) pmol/L	14.7	11.8-20.8	2.5	13.7-15.6

Triiodothyronin (T3) nmol/L	2.6	2.1-3.5	0.4	2.5-2.7

Thyroxin (T4) nmol/L	108.6	75.0-165.0	22.2	100.1-117.1

Thyroxin-binding globulin (TBG) mg/L	361.4	220-650	96.7	324.6-398.6

Thyroid stimulating hormone (TSH) mU/L	1.75	0.42-4.5	0.98	1.38-2.12

Thyroid hormone metabolism parameters, means, ranges, standard deviation and 95% confidence interval.

Reference intervals: FT4: 9-22 ρmol/L, T4: 70-140nmol/L, T3: 1.2-3.0 nmol/L, TBG: 7-17 mg/L, TSH: 0.5-5.7 mU/L.

### Dl-PCBs

**Figure 1:**
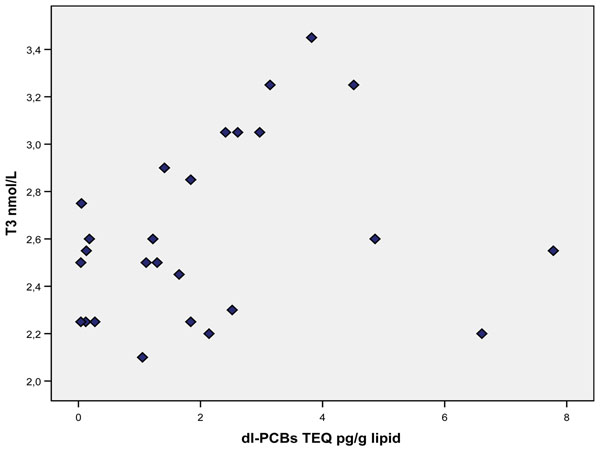
**Serum dl-PCBs and T3 levels (P=0.047) in the individuals at the age of 14-18 years** Serum dl-PCBs and T3 levels (P=0.047) in the individuals at the age of 14-18 years

A significant relationship was found between T3 and dl-PCBs (P=0.047, see figure 1). No relation was found with T4, FT4, TBG or TSH.

### PBDEs

**Figure 2:**
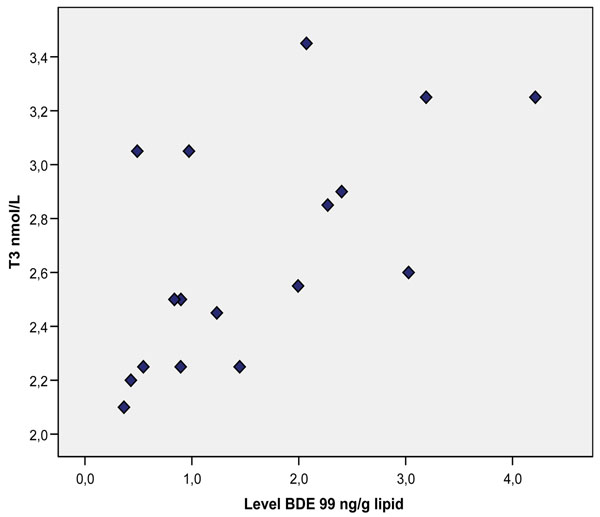
**Serum BDE-99 levels and T3 (P=0.003) in the individuals of the cohort at the age of 14-18 years.** Serum BDE-99 levels and T3 (P=0.003) in the individuals of the cohort at the age of 14-18 years.

Congener specific analysis revealed a positive correlation between BDE 99 and T3 (P=0.003, Figure 2), and with FT4 (P=0.048). For the ∑PBDEs no significant relationship was seen.

No association was found with T4, TBG or TSH levels.

## Discussion

In the current study a positive relation was seen between dl-PCBs and T3, and a positive relation with T3 and BDE 99 (see Figure 1 and 2). No relationship was seen with TBG.

In an earlier study of our cohort an increase in T4 and T4/TBG ratio was seen in children with higher dioxin levels in their breastmilk in the first and eleventh week postpartum [26]. The children now in their adolescence have normal serum TSH and T4 values.

TSH levels were significantly increased in relation to prenatal exposure at eleven weeks postpartum. None of these TSH-levels were above the WHO cut-off point of 5 mU/L, now also proposed to use as a biomarker for dioxin toxicity. However we have found effects on brain development in our cohort at the age of 8-12 years of age, while no baby had a TSH above 5 mU/L in their postnatal period [30] . Based on these outcomes it would seem a major error for governmental bodies to use a cut-off point of 5 mU/L as a biomarker for dioxin toxicity.

T3 is a product of the target organs. It looks as if the damage done by the two pollutants dl-PCBs and BDE 99 resulting in higher T3 levels takes place in the peripheral target organs perhaps by negatively influencing transporter proteins. It is widely known and accepted that human beings have effective compensation mechanisms. For instance the brain is capable of keeping T3 levels constant over a wide range (30-200 % of normal) of T4 levels by adapting the different deiodinases. However it is possible that with environmental pollutants transporter proteins are negatively influenced like for example the monocarboxylate transporter 8 (MCT 8), a protein necessary for the transport of T4 and T3 over the membrane into the cell in the hypothalamus, and when this transporter protein is lower or absent severe mental problems can arise as is seen in the Allan-Herndon-Dudley syndrome, a genetic MCT 8 deficiency, that is characterized by severe mental retardation and an increase in T3 but normal T4 and TSH [31]. In other words, a normal functioning pituitary gland and thyroid gland producing enough T4, does not exclude other organs having insufficient hormones due to hormone transporter problems.

Numerous studies have provided evidence that polyhalogenated aromatic hydrocarbons (PHAHs) and their metabolites affect the thyroid hormone system: 1) They may interfere directly with the thyroid gland, 2) with thyroid hormone metabolizing enzymes (uridine-diphosphate-glucuronyl transferases), iodothyronine deiodinases, and sulfotransferases which are located in the liver and the brain, 3) by interfering with the plasma transport system of the thyroid hormone by competing with plasma transthyretin (TTR) binding sites in animals, in humans this TTR is less important and TBG is the main transporter in plasma [32] and 4) influence membrane transporter proteins, that are specific for different target organs [33]. PBDEs have structural similarities to T3 and T4, therefore it has been hypothesized that PBDEs might interfere with the transport and metabolism of T3 and T4 [34]. Another possibility is the up-regulation of type 1 deiodinase, which is involved in the deiodination of T4 to T3 and reverse T3 [35].

Doucet [36] published a sharp increase in the content of PBDE’s in fetal livers of human fetuses after elective abortions in early to mid-gestation and in the placenta. Total PBDE’s increased over time from 284 ng/g lipid in 1998 to 1607 ng/g lipid in 2006. Main found significant higher PBDE-levels in the breastmilk of mothers whose newborn sons had cryptorchidism [37].

Effects on the thyroid homeostasis in relation to PBDEs have been seen in animal studies. In a study of mink ingesting PBDE via their feed, a decrease in T3 was seen [38]. A decrease in T3 and T4 was also seen in fetuses of pregnant sheep who were exposed to BDE-47 [39]. PBDEs have been shown to decrease T4 and free T4 in animals. T3 was also decreased in some studies, but to a lesser extent than total T4 [40,41] . Animals don’t have TBG like humans for the transport of T4 and T3 in blood.

In a study on 23 subjects living close to an electronic waste and 26 control, higher TSH levels were found in the studied subjects, who had higher PBDE levels in their serum compared to a control group (382 ng/g lipids versus 158 ng/g lipids) [42]. In a small study investigating 11 electronic dismantling workers, no significant effects were mentioned related to TSH, T3 or T4 [43].

No association of BDE-47 or PCB-153 with TSH or thyroid hormone concentrations were found in 110 men with high consumption of fish from the Baltic Sea [44]. In a later study of 182 females however, a relationship between PCB-153 concentrations in plasma and T3 levels was seen [45].

Thus, besides the remodelling effects of tissues by PBDE’s, as described by Main [37] in the form of cryptorchidism during prenatal life, health problems in later life are also possible and we speculate that these health problems are caused by effects on peripheral target organs, maybe through thyroid hormone transporter disruption. The background levels of the PBDE’s are rather high in the Netherlands compared to other European countries but still ten times lower than in the US. An enhanced effect due to multiple exposures to dl-PCBs as well might additively worsen the situation.

In conclusion, the thyroid hormone system in Dutch adolescents is influenced by current levels of dl-PCBs and PBDEs. Most plausible is a toxic effect in the peripheral target organs, involving the membrane transporter proteins. A disruption may be compensated, but it is questionable if this compensation is sufficient for all organs depending on thyroid hormones. Pathology in some target organs may be present. Quantification of this compensation is currently very difficult.

## Abbreviations

I-TEQ: International Toxic Equivalency factor; PCB: polychlorinated biphenyls; PCDD: polychlorinated dibenzo-p-dioxins; PCDF: furans = polychlorinated dibenzo-furans; PBDE: polybrominated diphenylether; HBCD: hexabromocyclododecane; TBBPA: tetrabromobisphenol A; DBDE: decabromodiphenyl ether; FT4: Free thyroxine; T3: triiodothyronine; T4: thyroxine; TBG: thyroxine-binding globulin; TSH: thyroid stimulating hormone; PCB: polychlorinated biphenyls; BDE 99: brominated diphenyl ether; MCT 8: monocarboxylate transporter 8

## Competing interests

The authors declare that they have no competing interests.

## Author’s contributions

ML, GtT, TV, WvA, TvT and JK were equally involved in the set-up and execution of the epidemiological part of the study, while ML and GtT should be considered as first authors. ML, KO and PdV were all involved in the study and performed the measurements of the chemicals. AB, MKvK, CM, HR and GC were involved in preparing the paper.
